# Increased Incidence of Premenstrual Syndrome in Females with Palmar Hyperhidrosis

**DOI:** 10.3390/ijerph18094697

**Published:** 2021-04-28

**Authors:** Chun-An Cheng, Yu-Cheng Liang, Yin-Han Chang, Chun-Gu Cheng, Chi-Hsiang Chung, Wu-Chien Chien

**Affiliations:** 1 Department of Neurology, Tri-Service General Hospital, National Defense Medical Center, Taipei 11490, Taiwan; cca@ndmctsgh.edu.tw; 2 Department of Information, Taiwan Fertilizer Company, Taipei 10457, Taiwan; travis0314@gmail.com; 3 Graduate Institute of Biomedical Informatics, Taipei Medical University, Taipei 11031, Taiwan; 4 Department of Psychology, National Taiwan University, Taipei 10621, Taiwan; caalice2003@yahoo.com.tw; 5 Department of Emergency Medicine, Taoyuan Armed Force General Hospital, National Defense Medical Center, Taoyuan 32549, Taiwan; 6 Department of Emergency Medicine, Tri-Service General Hospital, National Defense Medical Center, Taipei 11490, Taiwan; 7 Department of Emergency and Critical Medicine, Wan Fang Hospital, Taipei Medical University, Taipei 11696, Taiwan; 8 Department of Medical Research, Tri-Service General Hospital, National Defense Medical Center, Taipei 11490, Taiwan; g694810042@gmail.com; 9 School of Public Health, National Defense Medical Center, Taipei 11490, Taiwan; 10 Graduate Institute of Life Sciences, National Defense Medical Center, Taipei 11490, Taiwan

**Keywords:** autonomic dysfunction, premenstrual syndrome, palmar hyperhidrosis

## Abstract

Background: Premenstrual syndrome (PMS) is a common disorder affecting the quality of life of women of reproductive age. In a previous study, sex hormone imbalances and alterations in autonomic function were present in PMS, with parasympathetic dysfunction and sympathetic overactivity during the late luteal phase. Palmar hyperhidrosis (PH) presents with oversweating, heat and emotional stimulation, sympathetic hyperactivity and parasympathetic hypofunction. We hypothesized that the incidence of PMS is increased in females with PH. Methods: Data were retrieved from the Taiwanese National Health Insurance Database. The patients with PH were identified by the International Classification of Disease, 9th Revision, Clinical Modification (ICD-9-CM) disease code 780.8. Female patients matched by age and index day were used as the control group. The incidence of PMS was considered an outcome by the ICD-9-CM disease code 625.4. The factors related to PMS were analyzed by Cox regression. Results: The adjusted hazard ratio for the incidence of PMS was 1.276 (95% confidence interval: 1.05–1.488) in females with PH. Conclusions: This study found a positive correlation between PMS and female PH patients. Patients and physicians must understand the relationship of PMS with autonomic function alterations and other risk factors to prevent this problematic disorder.

## 1. Introduction

Premenstrual syndrome (PMS) is a common disorder in women during childbearing years. The symptoms include somatic and psychological symptoms that occur two weeks after ovulation and disappear after the start of the next menstrual cycle [1]. Sex hormone imbalance with low progesterone seems to affect the disease, but the autonomic system also makes some contributions. Past studies found lower parasympathetic function and sympathetic overactivity in the late luteal phase of the menstrual cycle [2]. The PMS symptoms of dizziness, presyncope, palpitation and nausea are related to hemodynamic conditions controlled by the autonomic system [3]. Many health problems share the same symptoms, such as depression and anxiety, chronic fatigue syndrome, irritable bowel disease and bladder pain, which are also associated with autonomic dysfunction [4,5].

Thermoregulation works by sensing warm or cold afferent changes to the preoptic hypothalamus, which then projects glutamine stimulation or gamma-aminobutyric acid GABAergic inhibition to the dorsomedial hypothalamus to enhance or reduce sweating by sympathetically affecting the raphe nucleus [6]. The sympathetic pathway involves stimulation of the ventromedial hypothalamus, which is regulated by emotions processed by the lateral nucleus of the amygdala or sensory pain that is processed by the anterior cingulate cores and periaqueductal grays with limbic system integration [7,8]. Then, excitation of the central sympathetic nucleus in the paramedian pontine reticular formation transports the message to synapse the upper thoracic sympathetic chain, supports sympathetic innervation and controls palmar sweating [9]. Palmar hyperhidrosis (PH) is thoracic sympathetic overactivity with oversweating of the hands during hot weather or stress, increasing the risk of skin infection [10]. Sympathetic and parasympathetic nerve functions work opposite to each other. Sympathetic hyperactivity with parasympathetic dysfunction in PH causes cardiovascular disease, ischemic stroke and gastroesophageal reflux [11,12,13,14,15]. Estrogen induces vasodilation and sweating [16]. Autonomic dysregulation seems to be similar between these two diseases, and the relationship between PMS and PH is worth evaluating.

The hypersympathetic and hypoparasympathetic functions of the autonomic system are present in PMS and PH. However, there is no evidence of an association between these two diseases. We performed a retrospective cohort study of female patients with PH and subsequent PMS episodes for 16 years.

We hypothesized that female patients with PH and underlying autonomic malfunction may have a long-term history of PMS. We also assessed other possible related factors to improve doctor and patient understanding of the comorbidities of PMS. The modification of parasympathetic function could improve autonomic dysfunction to reduce discomfort.

## 2. Materials and Methods

### 2.1. Database

In Taiwan, national health insurance was implemented 20 years ago, covering nearly all citizens. All medical institutions upload patient claim data to seek insurance reimbursement for healthcare services. The Longitudinal National Health Insurance Research Database (LNHIRD) contains one million randomly sampled patients. All outpatient records contain up to three outpatient International Classification of Disease, Ninth Revision, Clinical Modification (ICD-9-CM) codes, and inpatient records have up to five inpatient disease codes [17]. Our study assessed the occurrence of PMS in female patients with PH using the LNHIRD from 1 January 2000 to 31 December 2015. To protect patient privacy, unidentified ID numbers were provided. The obtained data include the patient’s age, sex, comorbidities and index dates of PMS and PH. Our study received ethical approval (TSGHIRB-B-110-05).

### 2.2. Design

**Figure 1:**
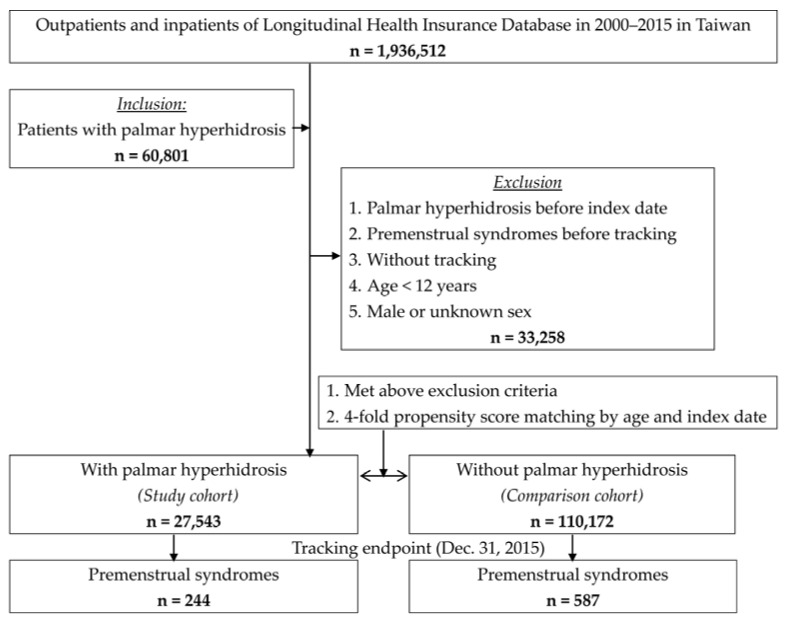
Flowchart of this study.

We identified newly diagnosed PH cases (ICD-9-CM disease code 780.8) from 1 January 2000 to 31 December 2015 in the Taiwanese LNHIRD. We evaluated the PH identified by ICD-9-CM diagnosis code 780.8 associated with major adverse cardiovascular events, ischemic stroke and gastroesophageal disease in previous studies [12,14,15]. The date of first visit for PH was assigned as the index date. We defined PMS (ICD-9-CM disease codes 625.4) as the outcome. The end date was the diagnosis of PMS in female patients with PH or the end time of the study. We excluded: (1) PH and PMS patients diagnosed before the study start date (1 January 2000); (2) patients younger than 12 years old (because they had not started menstruating); and (3) patients who were male or with an unidentified sex. The control group consisted of non-PH patients with four-folds matched by sex (female), age and index date meeting the exclusive criteria and then followed to the end of study. The study protocol is shown in Figure 1.

Comorbidities included obesity (ICD-9-CM code 278), depression (296.2–296.3, 296.82, 330.4, 331), anxiety (300.1–300.3, 300.5–300.9), thyrotoxicosis (242), fibromyalgia (729.1), hyperlipidemia (272), hypertension (401–405), diabetes mellitus (250), chronic kidney disease (580–589), asthma (493), chronic obstructive pulmonary disease (491, 492, 494, 496), abortion (630–639), pregnancy (640–677), irregular menstruation (626.4) and drinking (291,303, 305,571.0–571.4).

### 2.3. Statistical Analysis

The descriptive statistics were compared between groups using the chi-squared (*χ*^2^) test for categorical variables and Student’s *t*-test for continuous variables. Statistical significance was established as a *p* value < 0.05. The cumulative risk of PMS was stratified by PH or PH free with the log-rank test by Kaplan–Meier analysis. The risk factors for PMS were determined with a Cox proportional regression model according to the hazard ratio (HR). All analyses were performed using SPSS software version 21 (Asia Analytics Taiwan Ltd., Taipei, Taiwan).

## 3. Results

**Figure 2:**
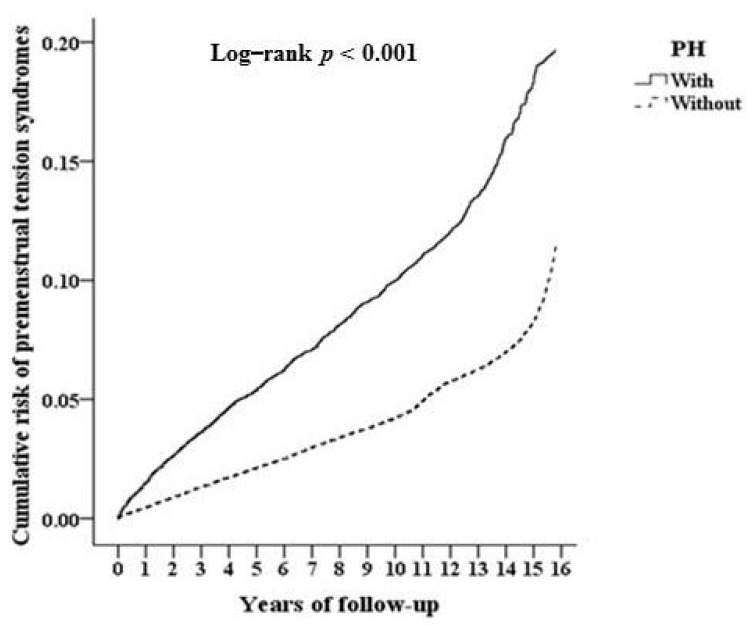
Kaplan–Meier analysis of the cumulative risk of premenstrual syndrome stratified by PH with the log-rank test.

The incidence of PMS was tracked for a mean of 8.9 years of follow-up. The mean age of PH patients was 28.3 years old (range 12–72). The occurrence of PMS was significantly higher in the PH group (0.88%, 244/27,543) than in the control group (0.53%, 587/110,172) (log-rank *p* < 0.001, Figure 2).

The occurrence of PMS was higher in the PH group than in the control group and associated with depression, anxiety, hyperlipidemia, hypertension, diabetes mellitus, living in western Taiwan and more densely populated urban areas and seeking medical treatment. However, fewer comorbid conditions, including abortion, pregnancy, irregular menstruation, chronic kidney disease and drinking, were noted in PH patients. Percentages of thyrotoxicosis, fibromyalgia, asthma, chronic obstructive pulmonary disease and season were similar in both groups (Table 1).

The risk factors for PMS were more numerous in PH patients, with an HR of 1.276 (95% confidence interval (CI): 1.05–1.488), and included obesity (HR: 1.386 (95% CI: 1.052–1.764)), depression (HR: 2.01 (95% CI: 1.726–3.348)), anxiety (HR: 2.154 (95% CI: 1.705–3.492)), hyperlipidemia (HR: 1.529 (95% CI: 1.284–2.03)), hypertension (HR: 1.443 (95% CI: 1.156–1.911)), diabetes mellitus (HR: 1.486 (95% CI: 1.209–1.976)), chronic kidney disease (HR: 1.197 (95% CI: 1.01–1.359)) and irregular menstruation (HR: 2.121 (95% CI: 1.573–4.009)), after adjustment for other factors (Table 2).

The stratified analysis of various variables showed that the occurrence of PMS in the PH group compared with the PH free group. After adjusting other factors, the risk of PMS for the patients with PH versus PH free was adjusted HR 1.276 (*p* = 0.002). The risk of PMS for the patients with diabetes mellitus was adjusted HR 1.301 (*p*< 0.001); the risk of PMS for the patients with anxiety was adjusted HR 1.286 (*p*< 0.001); the adjusted HR of PMS was 3.321 in the patients with obesity (*p* < 0.001); the adjusted HR of PMS was 7.411 (*p* < 0.001) in the patients with thyrotoxicosis; and the adjusted HR of PMS was 3.568 in the patients with fibromyalgia while PH group compared with PH free group (*p* < 0.001) (Table 3).

## 4. Discussion

This is the first survey on the risk of PMS in female PH patients. Female PH patients with excessive palmar sweating and sympathetic hyperactivity combined with parasympathetic dysfunction had a higher risk of PMS. The mechanism of PMS is decreased progesterone, serotonin impairment, central GABA dysfunction and decreased parasympathetic function combined with hypersympathetic dysfunction in the late luteal phase of the menstrual cycle, causing somatic and psychological syndromes [2,18]. This study enhances understanding of the shared autonomic dysfunction in PMS and PH.

PMS affects up to 12% of women in France. PMS with psychological conditions such as anxiety or depression is called premenstrual dysphonic syndrome (PMDD). There are fewer PMDD patients with a prevalence of 1.3– 5.3% [1]. The ovaries are innervated by the upper lumbar sympathetic and sacral parasympathetic nerves. The cell bodies of the pelvic splanchnic nerve are located at T12-L1 and exit through the sacral foramina of the sacral parasympathetic nerve. A previous study found low norepinephrine levels in PMS due to fluid overload, low baroreflex sensitivity and higher low-frequency blood pressure variability during the late luteal phase of the menstrual cycle after a postural challenge [19]. These findings indicate postural sympathetic overactivity related to blood pressure control in PMS. PMDD is indicated by psychological symptoms with sympathetic hyperactivity and higher norepinephrine concentrations and needs to be treated with antipsychotic agents. Parasympathetic function is markedly reduced in PMDD during the follicular and late luteal phases compared with that in PMS during the late luteal phase of the menstrual cycle [2].

Female PH patients with PMS who are living in areas with greater urbanization and who have greater emotional stress exhibit stimulation of the amygdala nucleus through the hypothalamus–pituitary gland–adrenal gland axis and increased sympathetic neurotransmission [20]. They seek higher levels of healthcare and demonstrate greater concern of their health. Reproductive-age women with pregnancy or abortion have lower incidences of PMS; they stop menstruating with progressive increase of estrogen and progesterone after pregnancy, which is a potential reason the women visited obstetric section without PMS diagnosis from the gynecology section. The heart rate variability (HRV) is measured to assess autonomic function by heart rate interval spectral analysis [21]. Mental disorders, including anxiety and depression, were associated with reduced HRV and a higher risk of cardiovascular disease in a meta-analysis [4]. High-frequency HRV reduction is shown in PMS with depression [22]. College students have more negative health subjective perceptions and stress associated with the intensity of PMS [23]. Our study found that anxiety or depression increased the risk of PMS two-fold. Obese patients have sympathetic overactivity with obstructive sleep apnea and exercise intolerance [24]. University students who smoke and have high calorie/fat/sugar/salt food consumption have high prevalence of PMS [25]. This study observed an increased incidence of PMS in the obesity population. Hypertension patients presented higher sympathetic arousal and increased inflammation promoted atherosclerosis [26]. The PMS patients also had higher prevalence of hypertension in a past study [27]. A study of diabetes mellitus in young people found decreased total HRV, high frequency HRV and increased low-frequency HRV in diabetes mellitus patients compared with nondiabetic controls [28]. Our study showed that young female patients with hypertension or diabetes mellitus had an increased incidence of PMS. Patients with chronic kidney disease have fluid overload with parasympathetic impairment and higher sympathetic activity [29], and our study showed chronic kidney disease increases the risk of PMS. Thyrotoxicosis and fibromyalgia presented hypersympathetic activity [30,31], higher parasympathetic activity in asthma, and lower parasympathetic function in chronic obstructive pulmonary disease [32,33] in past studies unrelated to PMS; the potential reasons might be that, in patients with chronic obstructive pulmonary disease retrieved by diagnosis codes, suitable treatments were performed after diagnosis confirmation in claim dataset. The lower prevalence of thyrotoxicosis and fibromyalgia were noted but in the statistical analyses they were not significantly different, and PMS occurred in the late luteal phase of menstrual cycles without season effect.

Treatment strategies relieve the symptoms during the late luteal phase of the menstrual cycle. Lifestyle modifications involve avoidance of a high salt diet, caffeine, smoking and drinking, emotional support and adequate exercise [1]. Pain symptoms can be relieved by nonsteroidal anti-inflammatory drugs. Contraceptive pills can maintain a steady level of sex hormones during the menstrual cycle, which can address the lower progesterone levels in the late luteal phase of the menstrual cycle. The use of hormonal contraception reduced the intensity of PMS in a past study [34]. Gonadotropin-releasing hormone-inhibited ovarian function can improve PMS with a risk of cardiovascular and osteoporosis with short-term use; diuretics reduce edema with bloating and breast discomfort. The symptoms of depression and anxiety require antidepressants and anti-anxiety medicines. Calcium and vitamin D may be useful in some PMS patients [35]. Kampo therapy and yoga may improve vascular recovery and reverse autonomic dysfunction in PMS patients [36,37] In previous studies, endoscopic thoracic sympathectomy decreased the sympathetic activity of the thoracic chain with relatively increased cardiac parasympathetic activity for PH, with a cardioprotective effect [38], but it had no vagal effect on gastroesophageal reflux disease [39]. However, we investigated endoscopic thoracic sympathectomy by ICD-9-CM code 05.29, and this procedure was not associated with a reduced incidence of PMS, with an adjusted HR 1.058 (95% CI: 0.769–1.454); a potential reason may be that 84% of patients compensated oversweating in the lower limbs with thoracic cardiac sympathetic function reductions with compensatory lumbar chain sympathetic hyperactivity and no changes in sacral parasympathetic activity stimulation [40]. Acute exercise increases sympathetic activity, but physical training could increase parasympathetic tone during the recovery phase [41]. Aerobic exercise increases parasympathetic function in healthy subjects, and the effect of aerobic exercise on reversing autonomic dysfunction in PMS is worthy of future evaluation.

The strength of the present study was that it assessed the association between PH and PMS in a large sample using a population-based design. However, some limitations do exist in our study. First, because we used disease codes for PMS, the lack of symptom score surveys meant that information on severity was unavailable. This would confirm different intensities of PMS in female PH patients. Second, Chinese women are shy and seek healthcare less frequently compared with Western countries, taking medicine by themselves because of the traditional culture. Meanwhile, the symptoms get worse, to the point of disturbing their lives, and then they or their families seek medical help. A lower prevalence of the two diseases was present in young females seeking healthcare according to the claim dataset; thus, a registration study is needed. Third, oversweating patients in Taiwan, located in a subtropical area, seek healthcare for PH more than those in Western countries. Under global warming, the residents of other countries may increase the prevalence of sweating; the sequent PMS is an issue worth noting. Our study surveyed patients of Chinese ethnicity; the generalizability needs additional study in the future to confirm.

## 5. Conclusions

To our knowledge, this is the first paper on the association of females with PH and PMS. We extend the literature on the sympathovagal imbalance in PMS that may be induced in female patients with PH, with similar autonomic dysregulation. Our findings indicate an increased risk of PMS in female patients with PH. Patients with severe PMS should consider consulting a gynecologist or psychiatrist for treatment. According to this study, which noted the same autonomic dysfunction in these two diseases, aerobic exercise with parasympathetic revision may affect the underlying autonomic imbalance and needs future confirmation.

## Figures and Tables

**Table 1:** Characteristics of the study cohort at baseline.

	Palmar Hyperhidrosis (27543)	Control (110172)	*p*
Age (years)	28.30 ± 13.23	28.32 ± 13.25	0.823
Obesity	246 (0.89%)	1014 (0.92%)	0.697
Depression	5344 (19.4%)	19187 (17.42%)	<0.001 *
Anxiety	5672 (20.59%)	20013 (18.17%)	<0.001 *
Thyrotoxicosis	39 (0.14%)	168 (0.15%)	0.676
Fibromyalgia	42 (0.15%)	180 (0.16%)	0.687
Hyperlipidemia	4752 (17.25%)	18245 (16.56%)	0.006 *
Hypertension	5711 (20.73%)	21159 (19.21%)	<0.001 *
Diabetes mellitus	5523 (20.05%)	19887 (18.05%)	<0.001 *
Chronic kidney disease	2975 (10.8%)	12797 (11.62%)	<0.001 *
Asthma	2480 (9%)	9899 (8.99%)	0.921
Chronic obstructive pulmonary disease	3016 (10.95%)	12007 (10.9%)	0.805
Abortion	42 (0.15%)	1782 (1.62%)	<0.001 *
Pregnancy	1678 (6.09%)	16782 (15.23%)	<0.001 *
Irregular menstruation	1588 (5.77%)	6725 (6.1%)	0.035 *
Drinking	1604 (5.82%)	6781 (6.15%)	0.04 *
Season			0.999
Spring (March–May)	5022 (18.23%)	20088 (18.23%)	
Summer (June–August)	6731 (24.44%)	26924 (24.4%)	
Autumn (September–November)	7782 (28.25%)	31128 (28.25%)	
Winter (December–February)	8008 (29.07%)	32032 (29.07%)	
Location			<0.001 *
Northern Taiwan	9215 (33.46%)	28561 (25.92%)	
Middle Taiwan	7203 (26.15%)	26184 (23.77%)	
Southern Taiwan	7675 (27.87%)	27354 (24.83%)	
Eastern Taiwan	3429 (12.45%)	19446 (17.65%)	
Outlets islands	21 (0.08%)	8627 (7.83%)	
Urbanization level			<0.001 *
1 (The highest)	8216 (29.83%)	28901 (26.23%)	
2	9034 (32.8%)	35121 (31.88%)	
3	4125 (14.98%)	21443 (19.46%)	
4 (The lowest)	6168 (22.39%)	24707 (22.43%)	
Levels of healthcare			<0.001 *
Medical center	13309 (48.32%)	33452 (30.36%)	
Regional hospital	8245 (29.94%)	40267 (36.55%)	
Local hospital	5989 (21.74%)	36453 (33.09%)	

* *p* < 0.05.

**Table 2:** Factors of premenstrual syndrome.

Variables	Crude Hazard Ratio (95% Confidence Interval)	*p*	Adjusted Hazard Ratio (95% Confidence Interval)	*p*
Palmar hyperhidrosis	1.389 (1.186–1.673)	<0.001 *	1.276 (1.05–1.488)	0.002 *
Age	1.065 (0.864–1.372)	0.786	0.975 (0.725–1.211)	0.894
Obesity	1.599 (1.182–1.87)	<0.001 *	1.386 (1.052–1.764)	<0.001 *
Depression	2.586 (1.875–3.783)	<0.001 *	2.01 (1.726–3.348)	<0.001 *
Anxiety	2.677 (1.894–3.86)	<0.001 *	2.154 (1.705–3.492)	<0.001 *
Hyperlipidemia	1.798 (1.482–2.403)	<0.001 *	1.529 (1.284–2.03)	<0.001 *
Hypertension	1.701 (1.385–2.311)	<0.001 *	1.443 (1.156–1.911)	<0.001 *
Diabetes mellitus	1.725 (1.406–2.375)	<0.001 *	1.486 (1.209–1.976)	<0.001 *
Chronic kidney disease	1.286 (1.095–1.422)	<0.001 *	1.197 (1.01–1.359)	0.039 *
Irregular menstruation	2.989 (1.762–7.264)	<0.001 *	2.121 (1.573–4.009)	<0.001 *
Abortion	1.623 (1.024–2.765)	0.025 *	1.307 (0.862–1.872)	0.184
Thyrotoxicosis	2.289 (0.278–5.24)	0.909	2.001 (0.185–2.701)	0.928
Fibromyalgia	3.454 (0.425–6.187)	0.918	2.271 (0.193–4.229)	0.937
Asthma	1.451 (0.896–1.782)	0.286	1.186 (0.715–1.607)	0.38
Chronic obstructive pulmonary disease	1.209 (0.776–1.509)	0.335	1.078 (0.622–1.403)	0.478
Drinking	1.592 (0.751–2.248)	0.375	1.57 (0.724–2.188)	0.392
Season				
Spring	Reference		Reference	
Summer	1.185 (0.789–1.506)	0.289	1.086 (0.725–1.348)	0.31
Autumn	1.209 (0.813–1.584)	0.178	1.113 (0.774–1.387)	0.287
Winter	1.288 (0.856–1.617)	0.144	1.187 (0.798–1.401)	0.209
Location				
Northern Taiwan	Reference		Multicollinearity with urbanization level	
Middle Taiwan	0.903 (0.397–1.713)	0.629		
Southern Taiwan	0.984 (0.441–1.786)	0.583		
Eastern Taiwan	0.845 (0.342–1.684)	0.675		
Outlets islands	5.254 (0.013–278.901)	0.996		
Urbanization level				
1 (The highest)	1.986 (1.5–2.976)	<0.001 *	1.875 (1.357–2.78)	<0.001 *
2	1.624 (1.184–2.773)	<0.001 *	1.523 (1.01202.622)	0.038 *
3	1.301 (0.905–2.501)	0.099	1.112 (0.787–2.403)	0.248
4 (The lowest)	Reference		Reference	
Levels of healthcare				
Medical center	1.682 (1.249–2.187)	<0.001 *	1.572 (1.172–2.08)	<0.001 *
Regional hospital	1.603 (1.24–2.113)	<0.001 *	1.533 (1.159–2.001)	<0.001 *
Local hospital	Reference		Reference	

* *p* < 0.05.

**Table 3:** Factors of premenstrual syndromes stratified by variables by using Cox regression.

PH	with			without (Reference)			with vs. without (Reference)	
Stratified	Events	PYs	Rate (Per 10^5^ PYs)	Events	PYs	Rate (Per 10^5^ PYs)	Adjusted Hazard Ratio (95% Confidence Interval)	*p*
**Total**	244	243,198.75	100.33	587	982,035.11	59.77	1.276 (1.05–1.488)	0.002 *
**Abortion**								
Without	231	242,677.79	95.19	406	965,437.89	42.05	1.221 (1.036–1.407)	0.015 *
With	13	520.96	2,495.41	181	16,597.22	1090.54	1.74 (1.431–2.029)	<0.001 *
**Pregnancy**								
Without	244	228,338.22	106.86	587	806,427.21	72.79	1.276 (1.05–1.488)	0.002 *
With	0	14,860.53	0.00	0	175,607.90	0.00	–	-
**Irregular menstruation**								
Without	114	228,973.97	49.79	303	921,235.07	32.89	1.152 (1.007–1.342)	0.044 *
With	130	14,224.78	913.90	284	60,800.04	467.10	1.487 (1.249–1.739)	<0.001 *
**Obesity**								
Without	185	240,576.30	76.90	537	972,247.92	55.23	1.058 (0.876–1.239)	0.176
With	59	2,622.45	2,249.81	50	9,787.19	510.87	3.321(2.735–3.918)	<0.001 *
**Depression**								
Without	135	194,652.60	69.35	344	803,975.95	42.79	1.232 (1.036–1.433)	0.015 *
With	109	48,546.15	224.53	243	178,059.16	136.47	1.297 (1.145–1.598)	<0.001 *
**Anxiety**								
Without	130	191,977.17	67.72	336	797,121.35	42.15	1.229 (1.024–1.428)	0.025 *
With	114	51,221.58	222.56	251	184,913.76	135.74	1.286 (1.067–1.577)	<0.001 *
**Thyrotoxicosis**								
Without	239	242,810.24	98.43	585	980,519.79	59.66	1.254 (1.023–1.438)	0.025 *
With	5	388.51	1286.97	2	1515.32	131.99	7.411 (5.986–8.63)	<0.001 *
**Fibromyalgia**								
Without	239	242,757.26	98.45	583	980,377.17	59.47	1.259 (1.038–1.482)	0.011 *
With	5	441.49	1132.53	4	1657.94	241.26	3.568 (2.937–4.161)	<0.001 *
**Hyperlipidemia**								
Without	166	200,789.30	82.67	399	805,723.03	49.52	1.267 (1.048–1.48)	0.003 *
With	78	42,409.45	183.92	188	176,312.08	106.63	1.315 (1.079–1.529)	<0.001 *
**Hypertension**								
Without	161	191,641.64	84.01	408	785,809.93	51.92	1.23 (1.021–1.431)	0.028 *
With	83	51,557.11	160.99	179	196,225.18	91.22	1.342 (1.104–1.568)	<0.001 *
**Diabetes mellitus**								
Without	164	193,743.13	84.65	411	800,312.44	51.35	1.253 (1.033–1.468)	0.016 *
With	80	49,455.62	161.76	176	181,722.67	96.85	1.301 (1.069–1.512)	<0.001 *
**Chronic kidney disease**								
Without	185	216,117.80	85.60	457	862,574.32	52.98	1.228 (1.012–1.432)	0.036 *
With	59	27,080.95	217.87	130	119,460.79	108.82	1.523 (1.297–1.804)	<0.001 *
**Asthma**								
Without	177	220,364.93	80.32	445	893,005.53	49.83	1.251 (1.008–1.429)	0.043 *
With	67	22,833.82	293.42	142	89,029.58	159.50	1.403 (1.189–1.635)	<0.001 *
**Chronic obstructive pulmonary disease**								
Without	181	215,499.72	83.99	449	871,425.58	51.52	1.239 (1.022–1.444)	0.029 *
With	63	27,699.03	227.44	138	110,609.53	124.76	1.389 (1.142–1.68)	<0.001 *
**Drinking**								
Without	189	228,294.07	82.79	476	921,110.28	51.68	1.225 (1.006–1.429)	0.043 *
With	55	14,904.68	369.01	111	60,924.83	182.19	1.549 (1.278–1.893)	<0.001 *
**Season**								
Spring	40	44,352.01	90.19	126	200,432.43	62.86	1.104 (0.893–1.278)	0.204
Summer	58	59,292.00	97.82	131	229,883.14	56.99	1.305 (1.042–1.514)	0.009 *
Autumn	67	68,792.85	97.39	148	262,712.13	56.34	1.314 (1.082–1.539)	<0.001 *
Winter	79	70,761.89	111.64	182	289,007.41	62.97	1.352 (1.109–1.586)	<0.001 *
**Urbanization level**								
1 (The highest)	72	72,713.27	99.02	103	264,744.44	38.91	1.935 (1.592–2.378)	<0.001 *
2	63	79,441.57	79.30	130	305,515.50	42.55	1.416 (1.163–1.672)	<0.001 *
3	47	37,447.12	125.51	164	190,805.68	85.95	1.111 (0.924–1.301)	0.199
4 (The lowest)	62	53,596.79	115.68	190	220,969.49	85.98	1.028 (0.84–1.194)	0.286
**Level of healthcare**								
Medical center	92	116,906.34	78.70	116	298,357.83	38.88	1.542 (1.289–1.801)	<0.001 *
Regional hospital	85	73,684.55	115.36	231	356,448.12	64.81	1.378 (1.136–1.587)	<0.001 *
Local hospital	67	52,607.86	127.36	240	327,229.16	73.34	1.32 (1.089–1.542)	<0.001 *

PYs, Person-years; * *p* < 0.05.

## Data Availability

Restrictions apply to the availability of these data. Data was obtained from National Health Insurance database and are available from the authors with the permission of National Health Insurance Administration of Taiwan.
